# The Influence of Alcohol on Rumination and Metacognitions in Major Depressive Disorder

**DOI:** 10.32872/cpe.5615

**Published:** 2022-12-22

**Authors:** Lana Gawron, Anna Pohl, Alexander L. Gerlach

**Affiliations:** 1 Institute of Clinical Psychology and Psychotherapy, University of Cologne, Cologne, Germany; Philipps-University of Marburg, Marburg, Germany

**Keywords:** major depressive disorder, rumination, metacognitions, alcohol consumption, self-medication, alcohol use disorder

## Abstract

**Background and Objectives:**

Comorbidity between major depressive disorder (MDD) and alcohol use disorder (AUD) is highly prevalent but reasons for this association are unclear. Rumination may activate metacognitive beliefs that contribute to the development and maintenance of rumination and depression. Negative metacognitions can further lead to other dysfunctional coping strategies (i.e., consumption of alcohol). We examined whether alcohol reduces (state) metacognitions, rumination and other disorder-specific processes in a group of individuals suffering from MDD.

**Method:**

In an experiment with three randomized conditions we investigated whether the consumption of alcohol, placebo or no alcohol (orange juice) affects (meta-)cognitions, depressive symptoms and / or psychophysiological variables while participants ruminate.

**Results:**

Voluntary rumination increased self-reported sadness, tension and rumination, tensed facial muscles and increased heart rate, but did not affect (state) metacognitions and heart rate variability. The consumption of alcohol did not influence rumination, metacognitions, depressive or psychophysiological measures.

**Limitations:**

We recruited a depressed population but excluded pathological alcohol use due to ethical considerations.

**Conclusions:**

We found no evidence that alcohol consumption affects rumination, metacognitions and other disorder-specific processes in MDD. However, rumination had a negative effect on various depression-specific processes, although it did not activate (negative state) metacognitions.

## Highlights

- The effect of alcohol on (meta-)cognitions, emotions, and psychophysiology was investigated with alcohol, placebo and a control group in MDD.
- No group differences were found both before and after alcohol consumption.
- Induced rumination did not activate (state) metacognitions, but affected various depression-specific processes.
- Future studies could activate metacognitions by providing false feedback about the controllability of such processes.

Rumination, the repetitive negative thinking about past events, possible causes and consequences of negative emotions (Nolen-Hoeksema, 1991), contributes to the development (e.g., Huffziger et al., 2009) as well as maintenance and severity of depressive episodes (e.g., Nolen-Hoeksema et al., 2008). Moreover, rumination has negative effects on somatic health, as illustrated by a number of psychophysiological changes such as decreased heart rate variability (HRV; e.g., Ottaviani et al., 2015), increased heart rates (HR; Ottaviani et al., 2016) and changes in muscular tension, e.g., in the corrugator EMG (Teasdale & Rezin, 1978).

According to the metacognitive model of rumination and depression (MCM), rumination is maintained by metacognitions reflecting on this type of perseverative thinking (Papageorgiou & Wells, 2003). Negative thoughts or other triggers initially activate positive metacognitive beliefs about the usefulness of rumination (e.g., “In order to understand my feelings of depression, I need to ruminate about my problems.”), and motivate further rumination. However, rumination prevents effective problem solving and intensifies negative affect. As a result, negative metacognitive beliefs emerge regarding the uncontrollability and harmfulness of rumination and its social consequences (e.g., “I cannot stop myself from ruminating.”; “People will reject me if I ruminate.”), thereby increasing the accessibility of negative and threatening information (e.g., negative thoughts or emotions), and thus exacerbating and maintaining depressive symptoms as well as promoting further rumination (Papageorgiou & Wells, 2004).

Both, clinical (e.g., Papageorgiou & Wells, 2003) and nonclinical studies (e.g., Solem et al., 2016) have shown that metacognitive beliefs about rumination are significant for the onset (Faissner et al., 2018; Papageorgiou & Wells, 2009) and maintenance (e.g., Solem et al., 2016) of depressive states / depression.

Negative metacognitions may also promote the use of dysfunctional behavioral strategies, such as the use of alcohol, to control or avoid recurrent negative thoughts. In the long term, however, these strategies may maintain negative metacognitions (cf. metacognitive model of generalized anxiety disorder; Wells, 2005; Wells, 2011). Although the MCM of generalized anxiety disorder focuses on worry and meta-worry, we assume that the assumptions regarding the use of other coping strategies can also be applied to the MCM for depression and rumination. Thus, we take a step beyond the original model by postulating that alcohol use functions as a cross-model coping strategy that can reduce rumination (see, e.g., Mollaahmetoglu et al., 2021) and possibly negative metacognitions (in the short term), making these thoughts and processes seem less uncontrollable and threatening.

According to the appraisal disruption model, alcohol can disrupt the appraisal of threatening information (i.e., cognitions; Sayette, 1993). More specifically, alcohol may interfere with the initial perception of stressful information by preventing negative memories and associated stressful concepts from being activated. Moreover, cognitive abstraction capacity is supposed to be reduced by alcohol (Sayette, 1993), which may also impede perseverative thinking and related metacognitions. Finally, when intoxication precedes a stressor, it can buffer the stress by attenuating appraisal, thereby protecting the person drinking from fully experiencing the stressor (Sayette, 2017). Applied to the context here, negative thoughts and processes promoted by metacognitions can also be defined as a type of threatening information whose appraisal can be attenuated by alcohol consumption. Furthermore, intoxication could prevent concepts associated with negative metacognitions, such as ruminative thoughts, from being activated, possibly leading to relief in terms of less threatening rumination or generally less aversive emotional states. Since this dysfunctional coping strategy is only helpful in the short term, alcohol may be consumed repeatedly in order to feel a facilitating effect (negative reinforcement). This could then lead to the development of a problematic drinking pattern or an alcohol use disorder (AUD).

Empirical evidence suggests that these negative metacognitions are in particular associated with problematic alcohol use (e.g., Spada et al., 2007). The higher the levels of maladaptive metacognitions are, the more likely alcohol is consumed in response to unpleasant aversive states (Moneta, 2011). In line with this, rumination is associated with alcohol consumption (e.g., Devynck et al., 2019) and with increased alcohol-related problems (e.g., Willem et al., 2011). In a group of individuals with risky consumption, the direct effects of alcohol on rumination and mood were examined and it was found that alcohol reduced rumination directly and also indirectly by changing mood (Mollaahmetoglu et al., 2021).

Apart from the study of Mollaahmetoglu et al. (2021), most empirical evidence for the association of rumination, depressed mood and alcohol use (disorder) is correlative (e.g., Heggeness et al., 2019). Moreover, these relationships have mostly been examined in analogue samples (e.g., Bravo et al., 2018), and metacognitions have been assessed as a *trait* variable (e.g., Faissner et al., 2018; Papageorgiou & Wells, 2009). However, it has been argued that mimicking typical problematic situations may also provoke the presence of *state*-dependent metacognitive beliefs about perseverative cognitions as well as their consequences, especially in clinical populations (Andor et al., 2008). Consistent with this, negative metacognitions following worrying, so negative state metacognitions, were more pronounced in patients with generalized anxiety disorder compared with control participants when they received feedback that indicated arousal while being asked to relax (Andor et al., 2008).

In light of previous findings, we believe it is important to examine the direct effects of alcohol consumption on perseverative cognitions, such as rumination, and negative state metacognitions in an experimental setting: indeed, if it is shown that people with depression can alter cognitive processes with the help of alcohol, this could provide a significant clue to the mechanisms underlying the high comorbidity of major depressive disorder (MDD) and AUD (e.g., Brière et al., 2014), with, for example, odds ratios between 2.0 (Kessler et al., 1997) and 3.8 (Grant & Harford, 1995).

Namely, depression-related cognitive / ruminative and metacognitive processes that appear uncontrollable and threatening may erroneously appear controllable and less threatening after alcohol consumption, which may be relieving in the short term, thus promoting further consumption and the development of AUD.

To our knowledge, no study has yet examined the direct effects of alcohol on negative (meta)cognitions and depression in a clinically depressed sample. Our aim was therefore to examine these effects on rumination and metacognition in MDD. We specifically focused on (negative) state metacognitions (cf. Andor et al., 2008). The negative appraisal of these state metacognitions may be interrupted by alcohol consumption and consequently appear less threatening (cf. Sayette, 1993). For a holistic understanding of the effects of alcohol on disorder-specific processes, we also wanted to investigate the influence of alcohol on emotional states and psychophysiology (heart rate, heart rate variability, muscle tension). According to some studies, alcohol can lead to an increase in heart rate (Weise et al., 1986), a reduction in HRV (Koskinen et al., 1994), and a decrease in muscle tension (Stockwell et al., 1982).

Our hypotheses were as follows: given that rumination has an unfavorable impact on negative affect and psychophysiology (see, e.g., Ottaviani et al., 2016), we hypothesized that (H1) induced rumination has a negative effect on sadness, tension, and on the extent of rumination itself, as well as on psychophysiological processes. We also hypothesized that (H2) alcohol consumption reduces rumination, (H3) alcohol consumption reduces negative state metacognitions about rumination that, according to the MCM of rumination and depression, should be triggered by induced rumination, and (H4) alcohol consumption reduces negative emotions such as sadness and experienced muscle tension intensified by rumination. Finally, in addition to rumination, alcohol consumption may also affect psychophysiology, although the direction of the effect in MDD is still unclear. We assumed an increase in HR and a decrease in HRV and muscle tension in individuals with depression (H5).

## Method

### Recruitment

Participants were recruited online (e.g., via facebook), with publicly distributed leaflets, posters and at the outpatient treatment center for psychotherapy. All participants received a compensation of 8.50 euros per hour and were offered counselling. Exclusion criteria were current or past substance use disorder or AUD, complete abstinence of alcohol, GAD, current use of psychoactive medication, liver damage, current or past psychotic episodes, and pregnancy. GAD was excluded to ensure that the main problem with repetitive negative content was rumination and not worrying. All participants signed an informed consent. The ethics committee of the German Psychological Association approved this study (SS 042017).

### Participants

Sixty-five participants (46 women) diagnosed with current MDD using a structured clinical interview (see Procedure) completed the study. Thirty-nine participants (40.5%) were diagnosed with additional comorbid disorders. Twenty-seven suffered from anxiety disorders (41.5%), ten from posttraumatic stress disorder (15.4%), three from obsessive compulsive disorder (4.6%), three from an eating disorder (4.6%), and five from somatic symptom disorders (7.7%). Sociodemographic data is presented in Table 1. Further characteristics can be found in Table A1 (Supplementary Materials). Power analyses according to G*Power 3 (Faul et al., 2007) indicated a required sample size of at least 54 participants, expecting a medium effect size f = .25 for the analysis of a repeated measures ANOVA (within-between interaction) at an alpha level of .05 and 95% power (cf. Andor et al., 2008; Stevens et al., 2017).

**Table 1:** Demographic Data of all Participants Separated by Group

Variable	AC (*n* = 22)	PC (*n* = 22)	OC (*n* = 21)
Mean Age (*SD*)	33.6 (11.5)	30.2 (11.8)	30.7 (12.9)
Sex, *n* (%)			
Women	15 (68.2)	16 (72.7)	15 (71.4)
Men	7 (31.8)	6 (27.3)	6 (28.6)
Education, *n* (%)			
O level	4 (18.2)	16 (72.7)	1 (4.8)
Specialized A level	1 (4.5)	3 (13.6)	6 (23.8)
A level	15 (68.2)	3 (13.6)	15 (71.4)
Still attending school	2 (9.1)	–	–
Family status, *n* (%)			
Unmarried	17 (77.3)	20 (90.9)	17 (81.0)
Married – living together	1 (4.5)	1 (4.5)	3 (14.3)
Divorced	3 (13.6)	1 (4.5)	–
Registered civil partners	–	–	1 (4.8)
Widowed	1 (4.5)	–	–
Treatment, *n* (%)			
Current outpatient treatment	4 (18.2)	2 (9.1)	4 (19.0)
Past outpatient treatment	16 (72.7)	15 (68.2)	12 (57.1)
Past psychiatric inpatient treatment	7 (31.8)	7 (31.8)	5 (23.9)
Past antidepressant medication	5 (22.6)	7 (31.8)	8 (38.1)

*Note.* AC = alcohol condition; PC = placebo condition; OC = control / orange juice condition. O level = ordinary level high school certificate; A level = advanced level high school certificate. The groups did not differ significantly.

### Procedure

**Figure 1:**
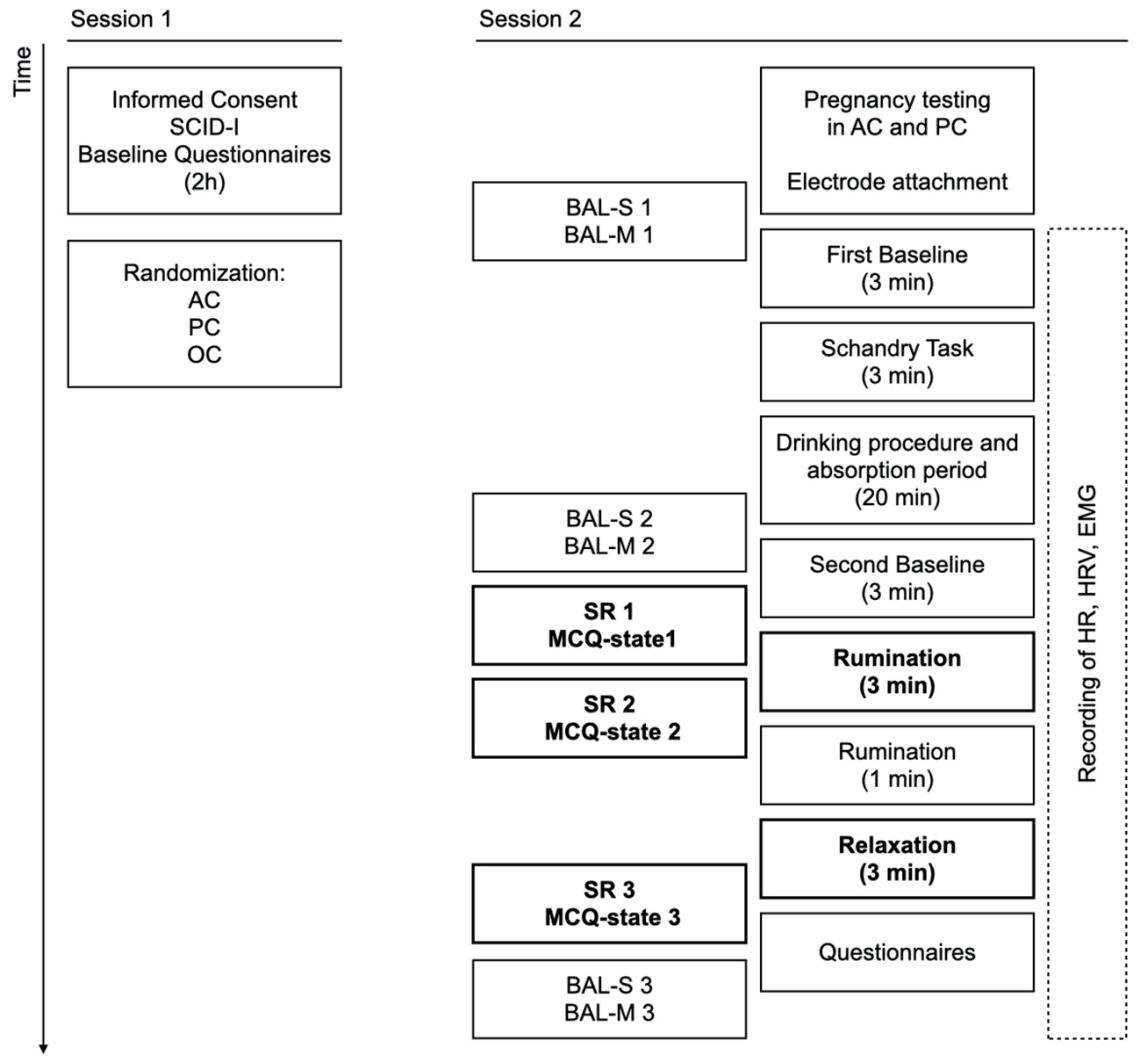
Procedure *Note.* Timing and overview of the two sessions. The blood alcohol level (BAL) was measured at the beginning, after the phase of drinking and at the end of the experiment. Self-reports (SR) and MCQ-state were assessed at three time points: before rumination, after rumination and after relaxation. An overview of all baseline questionnaires and all questionnaires used during the experiment can be found in section Measurements. AC = alcohol condition (*n* = 22); PC = placebo condition (*n* = 22); OC = control condition / orange juice (*n* = 21). BAL-S = participants' estimated BAL before each measurement of BAL; BAL-M = measured breath alcohol level. SR = self-reports, i.e., estimated levels of sadness, rumination, and tension. MCQ-state = two subscales of the Metacognitions Questionnaire, German version. HR = heart rate; HRV = heart rate variability; EMG = facial electromyography.

Participants were telephone screened and then received information about the experiment. They had to agree to participate in the study irrespective of whether they would receive alcohol or not. Participants with depressive symptoms were invited for a 2 h diagnostic session using the German version of the Structured Clinical Interview for the Diagnostic and Statistical Manual of Mental Disorders, 4^th^ version (SCID-I; Wittchen et al., 1997). A trained clinical psychologist conducted the interviews. Participants with MDD then completed several questionnaires (see Baseline Questionnaires) and were invited for a laboratory session. At this point, participants were fully randomized to three conditions (see Drinking Procedure). At the beginning of the laboratory session, electrodes for physiological measurement were attached and participants estimated their blood alcohol level (BAL). Then the BAL was measured. A three-minute resting period (first baseline) and an additional three-minute task (Schandry, 1981) followed, which will not be reported here. Then, a drinking phase of 15 minutes drinking and a five-minute break allowing for absorption of the alcohol followed. Participants again estimated their BAL and it was also measured. After a second three-minute resting period (second baseline), participants estimated their level of rumination, sadness and tension, and completed the state metacognitions questionnaire (MCQ-state; Andor et al., 2008). The *rumination induction procedure* (a variant of the worry induction procedure; Borkovec & Inz, 1990) followed. Participants were asked to write down three topics they regularly ruminated about and were to choose the currently most troubling one. They were then instructed to ruminate about this topic “like they normally did”. After ruminating for three minutes (rumination episode), participants reported their rumination, sadness and tension again and completed the MCQ-state. They were instructed to ruminate for another minute and then asked to relax for three minutes (relaxation episode). Following the relaxation, participants completed the self-reports and MCQ-state a third time as well as the WBSI, TCQ-R and CAS-I (see Questionnaires Used During the Experiment). In the end, they estimated their BAL and the BAL was measured one last time. After the experiment, participants were debriefed. The procedure is visualized in Figure 1.

### Drinking Procedure

All participants were asked to eat a light meal, specified in a handout, four hours prior to the experiment and to forego food and drinks containing caffeine from then on. They were requested to abstain from alcohol 24 hours prior to the experiment. Participants in the control condition (OC) were told that they would receive orange juice. Participants in the alcohol (AC) and placebo condition (PC) were both given the information that they would receive alcohol and that they would have to be picked up or wait until their BAL decreased below 0.3 ‰. All participants were tested at 4:00 pm. Female participants in the AC or PC were pregnancy tested. None of the participants tested positive. Finally, height and weight were measured.

Participants in the AC consumed a drink of 1:2 vodka and orange juice. Following a modified version of the Widmark formula, participant’s sex, weight, height and age was used to estimate the necessary amount of alcohol to reach a blood alcohol level of about 0.6 ‰ (Gerlach et al., 2006). The nonalcoholic beverage in the OC and PC was orange juice in comparable drinking quantity. In the PC, immediately before serving the beverages, a few milliliters of vodka were dropped on the orange juice and applied along the rims using a pipette (Stevens et al., 2014). Participants received three glasses with equal amounts of chilled beverage, each to be finished within five minutes. After drinking, participants waited five minutes.

Breath alcohol concentration was assessed by breathalyzer with an accuracy of +/- 0.03 mg/L (Dräger, Alcotest, 7410 plus). In the PC, the first measurement used a standard breathalyzer to ensure a BAL of zero. Then, a rigged breathalyzer with identical built was used giving a false feedback of 0.6 ‰ and then 0.7 ‰ BAL.

### Measurements

#### Baseline Questionnaires

##### Alcohol Use Disorder Identification Test (AUDIT)

The AUDIT (Dybek et al., 2006) is a brief screening scale developed by the World Health Organization (WHO) for early detection of problematic drinking. The original as well as the German version includes 10 questions regarding alcohol consumption, dependency symptoms and alcohol related problems. For each question, one of five statements related to alcohol use in the past year can be selected on a 5-point Likert-type scale ranging from 0 (“never”) to 4 (e.g., “daily or almost daily”). Cronbach’s α = .76.

##### Simplified Beck Depression Inventory (BDI-S)

The BDI-S (Schmitt et al., 2003) assesses current depressive symptoms with 20 items on a 6-point Likert-type scale ranging from 0 (“never”) to 5 (“almost always”), for example, “I feel sad.”. Cronbach’s α = .87.

##### Metacognitions Questionnaire 30 (MCQ-30)

The German version of the MCQ-30 (Arndt et al., 2011; a shortened version of the original Metacognitions Questionnaire; Cartwright-Hatton & Wells, 1997) is used to assess thoughts and beliefs (metacognitions) about worry. The questionnaire consists of five subscales (positive worry beliefs, beliefs about uncontrollability and danger, metacognitive efficiency, general negative beliefs, cognitive self-consciousness) assessed by 30 items (e.g., “Not being able to control my thoughts is a sign of weakness.”). Items/statements can be rated on 5-point Likert-type scales ranging from 1 (“not agree”) to 4 (“agree very much”). Cronbach’s α *=* .84.

##### Penn State Worry Questionnaire (PSWQ)

The German version of the PSWQ (Stöber, 1995) is a 16-item questionnaire assessing intensity, excessiveness and uncontrollability of worry (e.g., “I worry all the time.”) on a 5-point Likert-type scale ranging from 1 (“not at all typical of me”) to 5 (“very typical of me”). Cronbach’s α *=* .89.

##### Response Styles Questionnaire (RSQ)

The German version of the RSQ (Kühner & Weber, 1999) assesses people’s cognitive and behavioral strategies to cope with depressed mood with 32 items on 4-point Likert-type scales ranging from 1 (“almost never”) to 4 (“almost always”). The RSQ consists of the subscales rumination with 21 items (e.g., “When I am sad, I think about how sad I feel.”) and distraction with 11 items (e.g., “When I am sad, I go to my favorite place to get my mind off my feelings.”). Cronbach’s α *=* .69.

#### Questionnaires Used During the Experiment

##### Assessment of State Metacognitions (MCQ-state)

Since state-dependent changes in metacognitions can be assessed using the MCQ (cf. Andor et al., 2008), two subscales of the MCQ-30 (beliefs about uncontrollability and danger, general negative beliefs) were adapted to the experiential situation. An example is “My ruminating could make me go mad.”. Cronbach’s α *=* .97.

##### Rumination Score (RS)

The levels of sadness, tension and rumination were assessed on one rating scale each, ranging from zero (“absolutely not”) to 100 (“extremely so”) and then averaged. Cronbach’s α *=* .83.

##### White Bear Suppression Inventory (WBSI)

The German version of the WBSI (Fehm et al., 2000) measures thought suppression with 15 items (e.g., “There are things I prefer not to think about.”) on a 5-point Likert-type scale ranging from 1 (“strongly disagree”) to 5 (“strongly agree”). Cronbach’s α *=* .85.

##### Thought Control Questionnaire (TCQ)

The German version of the TCQ (Fehm & Hoyer, 2004) is a 30-item self-report measure assessing rumination, intrusive and unwanted thoughts. Items can be rated on 4-point Likert-type scales ranging from 1 (“never”) to 4 (“almost always”). Cronbach’s α *=* .67.

##### Cognitive Attentional Syndrome-Inventory (CAS-I)

The German version of the CAS-I (Wells, 2011) assesses maladaptive coping strategies (e.g., worrying, avoidance, use of alcohol/drugs) for dealing with negative thoughts, and negative and positive metacognitive beliefs. In total, the CAS-I consists of four questions. The first three questions are answered using a scale from 0 (“not at all”) to 8 (“all the time”) and refer to how much dealing with problems or worries about problems was done in the past week and how it was dealt with. The fourth question refers to positive and negative metacognitions, answered using a scale from 0 (“I do not believe in this belief at all.”) to 100 (“I am absolutely convinced that this belief is true.”). Cronbach’s α *=* .75.

### Psychophysiological Data Recording, Sampling and Analysis

Psychophysiological data (heart rate, respiration and facial muscle tension) were recorded using the Varioport (Becker Meditec, Karlsruhe, Germany). ECG was recorded at 512 Hz sample rate from three electrodes. The active electrodes were placed on the lowest left rib and on the right collarbone. Ground was affixed to the left collarbone. Respiration was assessed with a respiratory belt (128 Hz sample rate). Facial electromyography (EMG) was recorded in mV at 256 Hz sample rate over the *corrugator supercilii* on the left side of the face with two electrodes (TIGA-MED, Germany Ltd.). The EMG signal was preprocessed using an infinite impulse response high pass filter at 10 Hz. It was notch filtered at 50 Hz with a width of 3 Hz and rectified and smoothed using a two-step low pass filter with eight point moving average. For HRV, the root mean square successive differences (RMSSD) was calculated (cf. Task Force of the European Society of Cardiology and the North American Society of Pacing and Electrophysiology, 1996; Bertsch et al., 2012). Mean values were computed for each experimental 3-minute episode (baseline 2, rumination, relaxation).

### Data Analysis

Group differences concerning sociodemographic characteristics and self-reported BAL were tested using an ANOVA^1^1Initial exploratory analyses revealed a few outliers. However, there was no relevant change in the pattern of results when including vs. excluding outliers. Thus, results from the complete data set are reported. Deviations from the original data set are indicated in the data analysis (e.g., MCQ-state ratings). and Bonferroni-corrected post-hoc tests. Group differences concerning psychopathological variables (questionnaires) were analyzed using a MANOVA. A Pearson correlation was performed between problematic alcohol consumption (AUDIT) and the level of alcohol as a coping strategy (CAS-I). To test our hypotheses, we conducted several repeated measures ANOVAs^2^2The assumption of normality (ANOVA) or the equality of variances (repeated measures ANOVAs) was not met. Since the *F*-Test is relatively robust for violation of assumption (Finch, 2005; Tabachnick & Fidell, 2007), the ANOVA and the repeated measures ANOVAs were nevertheless conducted and results reported. Because the number of subjects varied across the variables, no repeated measures MANOVA could be calculated for the self-reports or for the biodata. Instead, several repeated measures ANOVAs were conducted with Bonferroni-corrected post-hoc tests. with Bonferroni-corrected post-hoc tests. Each ANOVA was analyzed by group (alcohol, placebo, orange juice).

To test H1 (rumination increases sadness, tension, rumination, and worsens psychophysiology) the measurement time points of all variables from “second baseline” to “rumination” were examined. H2 (alcohol reduces rumination) and H4 (alcohol reduces sadness and tension intensified by rumination) were tested in one model: for this, RS over time were analyzed. To test H3 (alcohol reduces negative state metacognitions), metacognitions ratings (MCQ-state) were analyzed. To test H5 (alcohol influences psychophysiology), EMG, HR and HRV over time were analyzed. In case sphericity was violated, the Greenhouse–Geisser adjustment was used.

## Results

### Manipulation Check

#### Coping Strategies

The correlation of AUDIT and CAS-I was significant (*r* = .46, *p* < .001). The most frequently used coping strategy was “to control emotions” (*M* = 6.0, *SD* = 2.0), followed by “the attempt not to think about anything” (*M* = 5.2, *SD* = 2.2), “to avoid situations” (*M* = 2.8, *SD* = 2.5), “to control symptoms” (*M* = 4.2, *SD* = 2.2), “to seek reassurance” (*M* = 3.5, *SD* = 2.3). The least used strategy was “to consume alcohol or drugs” (*M* = 2.5, *SD* = 2.0).

#### Self-Reported Alcohol Level and Measured Blood Alcohol Level

**Figure 2:**
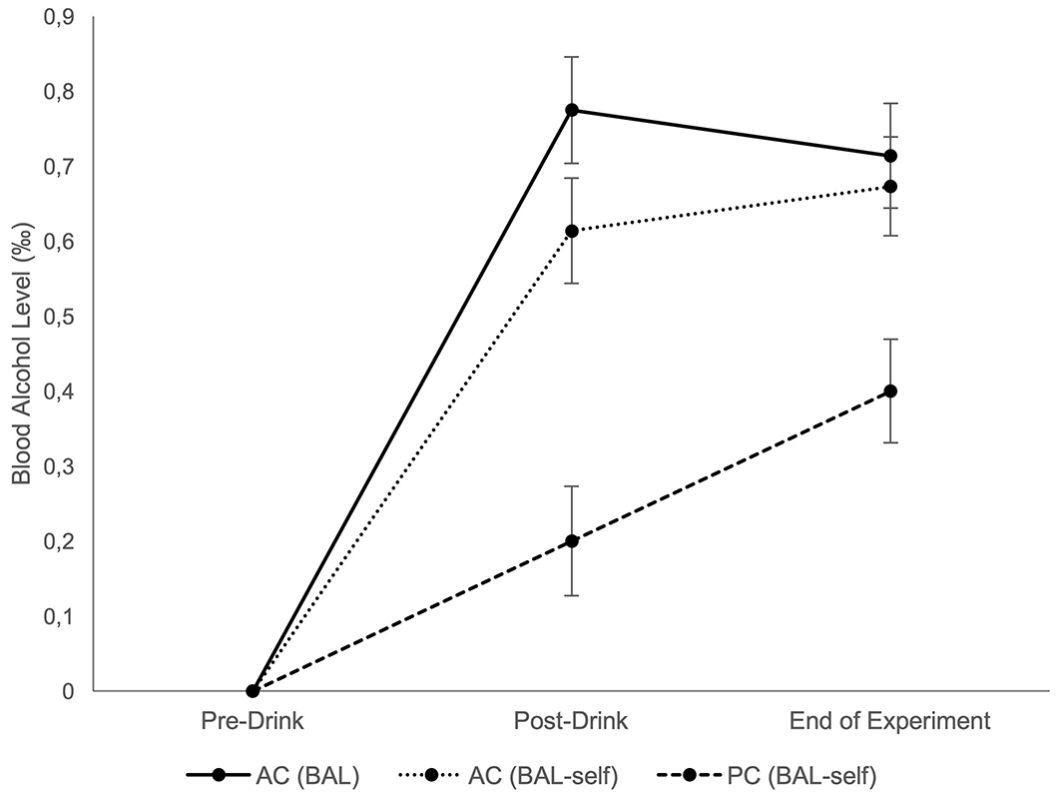
Blood Alcohol Level *Note.* Measured and estimated BAL. Data points represent values before and after the drinking procedure and at the end of the experiment; error bars depict 95% CI. AC = alcohol condition (*n* = 22); PC = placebo condition (*n* = 20). BAL = measured breath alcohol level in AC; BAL-self = participants' estimated BAL before each measurement of BAL. Control condition is not included.

Compared to baseline, in both AC and PC self-estimated alcohol levels (in ‰) were higher after drinking (*M*_AC_ = 0.6, *SD* = 0.2, *M*_PC_ = 0.2, *SD* = 0.1) and after finishing the experiment (*M*_AC_ = 0.7, *SD* = 0.2, *M*_PC_ = 0.4, *SD* = 0.2). The manipulation in the PC can be considered successful: 20 of 22 participants believed that they had been given alcohol. Two subjects (PCs) were excluded because their self-estimated BAL was 0.0 ‰ at all measurement points and then assigned to the control condition for subsequent analyses. In the AC, the measured BAL was 0.8 ‰ (*SD* = 0.2) after the drinking period and 0.7 ‰ (*SD* = 0.2) at the end of the experiment (see Figure 2).

#### Rumination Induction Procedure (H1, H5)

**Figure 3:**
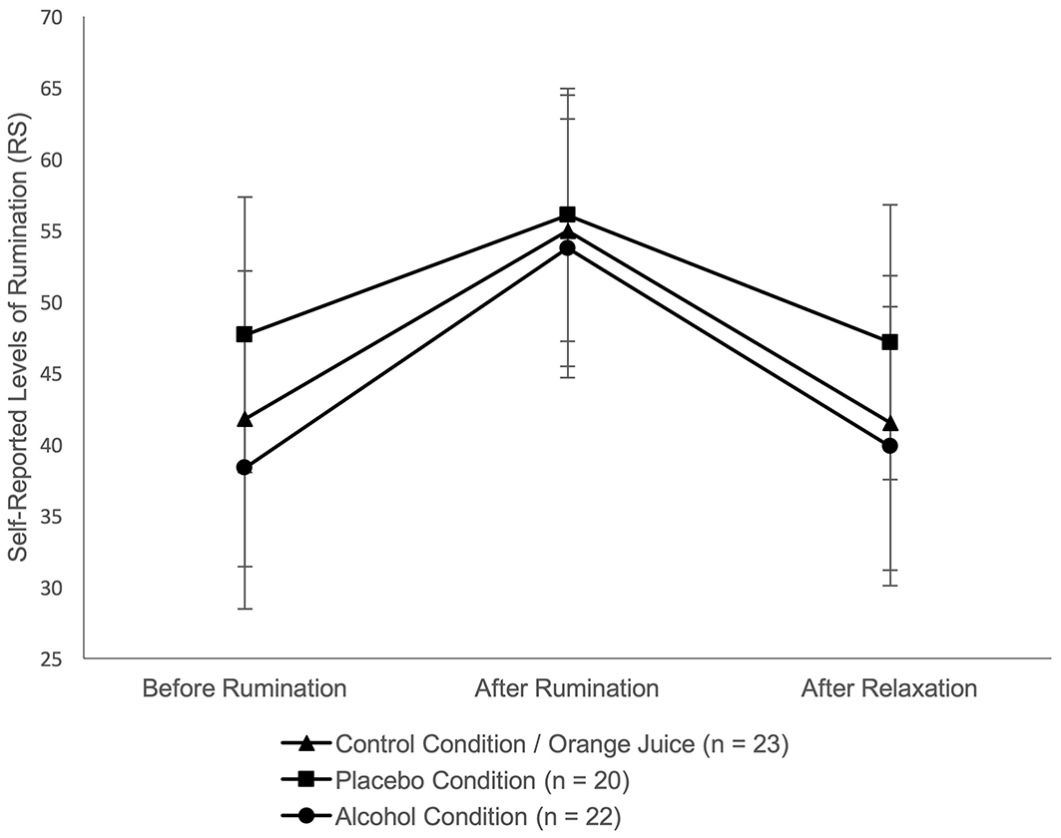
Results Over Time Separated by Group: a) Rumination Score *Note.* Data points represent the mean values before, after the rumination induction and after relaxation; error bars depict 95% CI. Estimates of depression (sadness, rumination, tension) were rated on a scale from 0 to 100.

Self-report: An initial univariate ANOVA revealed no significant group differences in the self-reports (*F*(2, 62) = .86, *p* = .427) and MCQ-state-ratings^3^3Since the first measuring time of the MCQ-ratings was subsequently integrated into the experiment, the repeated measures ANOVA was conducted with only *n* = 45. (*F*(2, 42) = .26, *p* = .772) before rumination induction. After rumination, RS were significantly higher (see Table 2 and Figure 3), whereas MCQ-state-ratings did not change (see Table 2).

**Figure 4:**
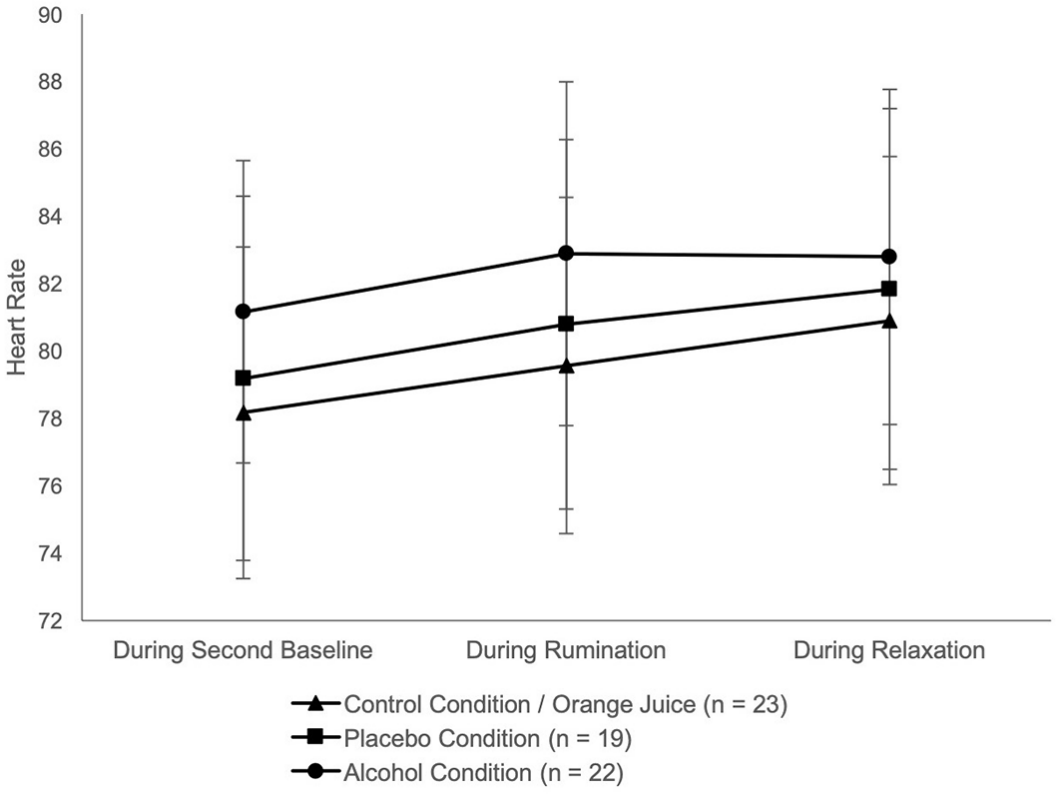
Results Over Time Separated by Group: b) Heart Rate *Note.* Data points represent the mean values of three time intervals: during second baseline, rumination and relaxation; error bars depict 95% CI.

**Figure 5:**
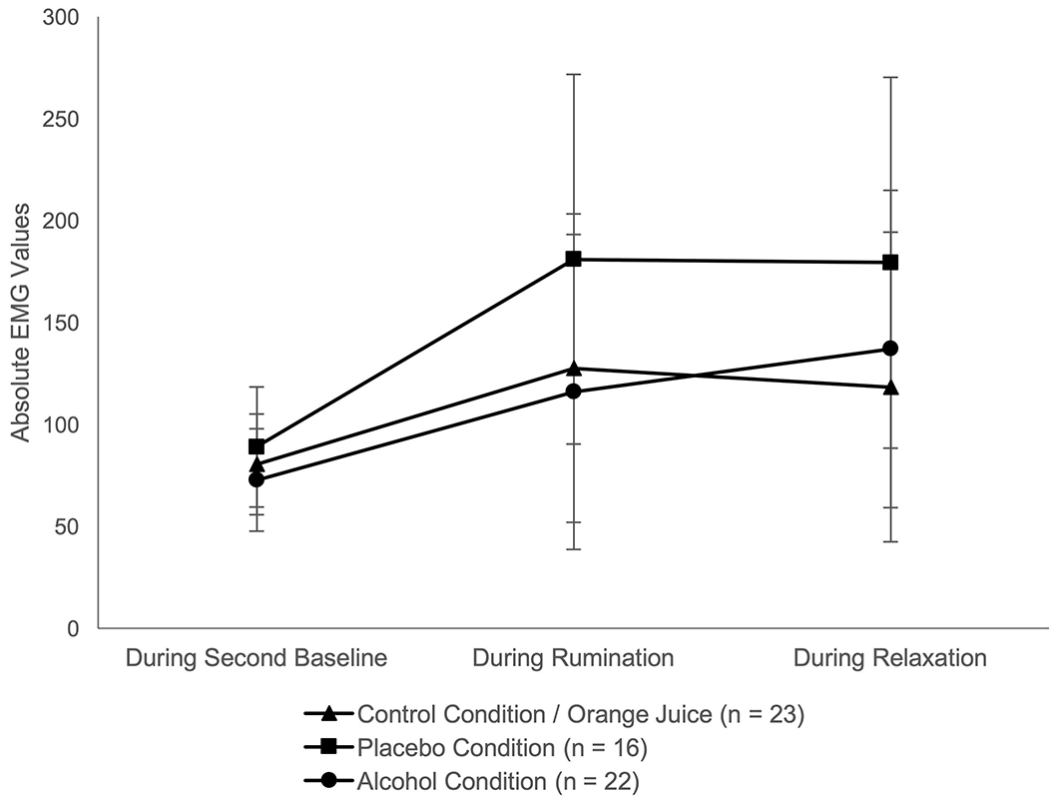
Results Over Time Separated by Group: c) EMG *Note.* Data points represent the mean values of three time intervals: during second baseline, rumination and relaxation; error bars depict 95% CI.

Psychophysiological measures: An initial univariate ANOVA^4^4Regarding EMG, three subjects (PC) were excluded from further analyses because they were identified as outliers in at least four of five relevant time intervals. Another subject was excluded because the recording of biodata failed. See Table A3 (Supplementary Materials) for an overview of all participants per condition. revealed no significant group differences in HR (*F*(2, 61) = .37, *p* = .690), HRV (*F*(2, 61) = 1.46, *p* = .240) or EMG (*F*(2, 58) = .36, *p* = .702) before rumination. HR and EMG increased significantly with rumination. Regarding HRV, there was no significant change in RMSSD during rumination or relaxation (see Table 2 and Figures 4, 5).

### Repeated Measures ANOVAs (H2-H5)

ANOVAs revealed a significant main effect of time for RS (*F*(1.52, 94.38) = 16.45, *p* < .001, η_p_ = .21), HR (*F*(2, 122) = 14.12, *p* < .001, η_p_ = .19), and EMG (*F*(2, 116) = 5.41, *p* = .006, η_p_ = .09). From “second baseline” (T1) to “rumination episode” (T2) there was a significant increase in RS, HR and EMG. From T2 to “relaxation” (T3) there was a significant decrease in RS (see Figure 3). From T2 to T3 there was no significant change in HR and EMG (see Figures 4, 5). No significant effect for group and no interaction effect for time × group was found in any variable (see Table 2 and A2, Supplementary Materials, for all significant and nonsignificant effects, Table A3, Supplementary Materials for mean values).

**Table 2:** Repeated Measures ANOVAs Results

Effects / Measures	*F*	*df*	*p*	$\eta_{p}^{2}$
Time				
RS	16.45	1.52, 94.38	< .001	.21
MCQ-state	.32	2, 84	*ns*	.01
HR	14.12	2, 122	< .001	.19
HRV	1.57	1.50, 91.26	*ns*	.03
EMG	5.41	2, 116	.006	.09
Group				
RS	.57	2, 62	*ns*	.02
MCQ-state	.31	2, 42	*ns*	.02
HR	.32	2, 61	*ns*	.01
HRV	1.08	2, 61	*ns*	.03
EMG	.74	2, 58	*ns*	.03
Time × Group				
RS	.38	3.05, 94.38	*ns*	.01
MCQ-state	.15	4, 84	*ns*	.01
HR	.56	4, 122	*ns*	.02
HRV	1.69	2.99, 91.26	*ns*	.05
EMG	.37	4, 116	*ns*	.01

*Note.* RS = Rumination score; MCQ-state = state version of the Metacognitions Questionnaire; HR = Heart rate, beats per minute (bpm); HRV = Heart rate variability, RMSSD; EMG = facial electromyography, absolute EMG values (uV). *ns* = nonsignificant.

## Discussion

We directly studied if alcohol affects disorder-specific processes in individuals suffering from MDD. In particular, we wanted to understand whether and how alcohol affects rumination and state metacognitions about rumination. In addition, we were interested in determining the extent to which rumination negatively affects other disorder-specific processes, such as intensifying sadness, and in terms of the MCM, is associated with negative metacognitions.

The rumination induction was successful: self-reported levels for rumination, tension, and sadness increased, as did HR and muscle tension. However, HRV and state metacognitions did not change. We were able to successfully establish a placebo condition (i.e., induce the belief of having consumed alcohol) in almost all participants. In addition, participants who reported higher alcohol consumption were more likely to report using alcohol for coping. Yet, alcohol use was the least reported coping strategy for aversive states in our sample.

In contrast to our first hypothesis, we did not find an increase in negative state metacognitions after rumination. It is possible that the type and implementation of the rumination induction procedure influenced our result. The procedure was originally developed for the induction of worry (Borkovec & Inz, 1990). Given, however, that worry and rumination are often transdiagnostically conceptualized as two forms of perseverative negative cognitions (e.g., McEvoy et al., 2013), the procedure for inducing rumination should have been sufficient to induce metacognitions about rumination, just as inducing worry was sufficient to induce metacognitions about worry (Andor et al., 2008). Yet, Andor and colleagues (2008) studied individuals with generalized anxiety disorder whose negative (trait) metacognitions are more pronounced than in individuals with MDD (Sun et al., 2017). Participants in the Andor study received false arousal feedback during the relaxation phase, making it more likely to experience worry and relaxation as uncontrollable. In other words, it was directly suggested to the participants in this study that their condition was not controllable. It is likely that both the type of disorder and the type of manipulation influenced the intensification of metacognitions. One approach for future studies might be to examine both state and trait metacognitions in relation to rumination and depressive symptomatology and to directly induce a sense of uncontrollability to participants.

However, another consideration against the background of the MCM is conceivable. In the Andor study as well as in our experiment, negative metacognitions were measured via two subscales of the MCQ-30. These scales assess the uncontrollability and danger of worry (reworded to rumination in our study), but not negative metacognitions with regard to social consequences of rumination, which, in terms of the MCM, are also typical for the perpetuation of depression. After successful induction, we did not find more pronounced metacognitions in terms of uncontrollability and danger, but we might have found changes in terms of metacognitions related to the social consequences of rumination. One way to measure both types of negative metacognitive beliefs about rumination would have been to include the Negative Beliefs About Rumination Scale (NBRS; Papageorgiou & Wells, 2001) in our experiment. In this way, we would have been even closer to the original model and the respective measurement methods (cf. Papageorgiou & Wells, 2003).

Also, it is possible that negative metacognitions do not need to be reinforced in certain situations to have a negative effect on perseverative thinking. It may be sufficient that these assumptions exist in the first place to maintain depressive states (e.g., Papageorgiou & Wells, 2009). If negative (state) metacognitions cannot be intensified even with the use of other experimental procedures, we nonetheless consider it advisable to reassess the long-term effects of negative metacognitions on the development of depression in a vulnerable group of participants. This would allow to further investigate the extent to which negative metacognitions are causal in the development and maintenance of depression.

Contrary to our hypotheses (H2-H5), we could neither show that alcohol consumption reduced experienced rumination, sadness, or muscle tension, nor that it reduced state metacognitions about rumination. The three groups did not differ regarding their RS nor in their ratings of metacognitions. There were also no differences between groups in terms of psychophysiological data. Alcohol did not change the negative effect of rumination on psychophysiological variables, nor did it increase physiological reactivity. Thus, surprisingly, we did not find evidence of alcohol effects on any process potentially relevant for the formation and maintenance of depression.

Conger (1956) suggested that alcohol may be used because it reduces muscular tension. However, alcohol did not reduce muscle tension nor change other measures of arousal. Whereas Conger’s notion can be found in many textbooks, the pharmacological (stress-reducing) effects of alcohol have only rarely been illustrated. According to a review of studies in social anxiety, for example, alcohol expectancy effects were more likely to be responsible for a reduction of aversive states such as anxiety than alcohol’s pharmacological properties (Battista et al., 2010). Thus, people who consume alcohol and expect a stress and tension-relieving effect, may experience such an effect regardless of pharmacological effects. Such positive alcohol expectancies should have been evident in both the AC and PC in comparison to the OC. Yet, in both self-reports and EMG the numerically highest values (indicating distress) were found in the PC. Since Conger's hypothesis refers mainly to anxiety-provoking situations, it should be noted that these assumptions may not apply in situations where other emotions, such as depression or sadness, are prominent. Or possibly, individuals might assume that alcohol is a helpful strategy, but notice when drinking that the strategy proves unsuccessful.

Significant positive correlations have previously been found between metacognitions and alcohol consumption as well as between anxiety, depression and alcohol consumption (Spada et al., 2007). The consumption of alcohol can therefore be regarded as a conscious strategy for dealing with aversive states (Quitkin et al., 1972). In the AC, however, alcohol consumption did not result in feeling less emotionally distressed than in the other two groups. Thus, we found no evidence that alcohol consumption reduces rumination, state metacognitions, or sadness in depressed individuals. Interestingly, our findings are consistent with those of a recent study on social anxiety, in which alcohol consumption had no attenuating effect on negative (post-event) rumination (Hagen et al., 2020), although consumption reduced (social) anxiety (Stevens et al., 2014). Mollaahmetoglu and colleagues (2021) found that alcohol had an effect on ruminative thoughts and mood at a low dose (about 0.2 mg/L) but not at a high dose (about 0.6 mg/L). It is therefore worth considering whether the desirable effects of alcohol in our study would also have been observed if we had used a lower dose. A promising approach for further studies could be to examine alcohol effects on rumination, metacognitions and depressive mood depending on the dose administered. Also, the question arises to what extent the model assumptions on alcohol effects (for a review see Sayette, 2017), which were investigated in the context of anxiety (disorders), can be transferred to other disorders and / or other emotional states, such as depression. It should be noted, however, that according to Sayette’s model (1993), appraisal disruption is expected only at higher levels of alcohol (i.e., at an amount of alcohol sufficient to cause cognitive impairment), and that we based our hypotheses on this model. Nonetheless, if alcohol may not be the usual choice for our participants, e.g., to control unpleasant cognitions, state metacognitions or emotions, it simply may not have this effect in the present sample due to selection bias. In order to ensure that alcohol is a preferred coping strategy, it would have been necessary to pre-screen, for example with the CAS-I (Wells, 2011).

Regarding the effects of alcohol consumption on (meta-)cognitive, emotional, and psychophysiological processes and its function in coping with depression, it can be stated that further research is needed to investigate these relationships in more detail.

### Limitations

One limitation of our study relates to the sample size, due to which only moderate effects could be detected. However, compared to the results of other clinical studies dealing with the effects of alcohol (e.g., in social anxiety disorder), the sample size we recruited can be considered sufficient (cf. Stevens et al., 2017). Another limitation relates to our procedure, which can be considered rather exploratory, as the direct effect of alcohol on state metacognitions has not been investigated before and therefore we could only assume that alcohol consumption may prevent negative state metacognitions from being appraised as threatening (cf. Sayette, 1993). In addition, it would have been helpful to assess the expected effects of alcohol on rumination or metacognitions before or during the experiment to include trait and actual expectancies of alcoholic effects into statistical analyses. A final limitation relates to the assessment of rumination. Here, for example, a rumination-related questionnaire with better psychometric properties may have been more suitable, (e.g., the Brief State Rumination Inventory; Marchetti et al., 2018).

### Conclusions

To our knowledge, this was the first study to directly examine the association between AUD and by assessing the effects of alcohol on rumination and state metacognitions in a sample of clinically depressed individuals. We did not find that alcohol reduced rumination, state metacognitions about rumination, or depressive symptoms. Thus, our results suggest that previous models of alcohol effects from the domain of anxiety disorders (e.g., Sayette, 1993) may not be easily transferable to the domain of depressive disorders.

Consistent with the findings of previous studies (see, e.g., Nolen-Hoeksema et al., 2008; Ottaviani et al., 2016), we were able to show that rumination negatively affects disorder-specific processes in MDD. Surprisingly, rumination did not elicit negative metacognitions about the uncontrollability and danger of rumination, although this would have been expected in terms of the MCM.

However, due to the novelty of this research approach, further studies are needed to further test existing models / theories linking depression and alcohol. For example, this could include studies with individuals who drink more and use alcohol more regularly for coping, with a modified paradigm, i.e., with other forms of rumination induction, with manipulated arousal feedback, or with a lower dose of administered alcohol, and / or with other (physiological) measurement methods.

## Supplementary Materials

The Supplementary Materials include the descriptive statistics of all questionnaires used, the results of the Bonferroni-Corrected Post-Hoc Tests for Repeated Measures ANOVAs, and the means of all measures across the three measurement time points (for access see Index of Supplementary Materials below).



GawronL.
PohlA.
GerlachA. L.
 (2022). Supplementary materials to "The influence of alcohol on rumination and metacognitions in major depressive disorder"
[Additional information]. PsychOpen. 10.23668/psycharchives.8391
PMC988112036762347

## Author Note

This study was part of the first author’s doctoral thesis (Gawron, 2022), which focused on the function of cognitive processes to explain the association of alcohol (consumption) and depression.
